# Tumor necrosis factor-α induces interleukin-34 expression through nuclear factor-κB activation in MC3T3-E1 osteoblastic cells

**DOI:** 10.3892/mmr.2014.2353

**Published:** 2014-06-25

**Authors:** YAQIONG YU, DI YANG, LIHONG QIU, HIROHIKO OKAMURA, JIAJIE GUO, TATSUJI HANEJI

**Affiliations:** 1 Department of Endodontics, School of Stomatology, China Medical University, Shenyang, Liaoning 110002, P.R. China; 2 Department of Histology and Oral Histology, Institute of Health Biosciences, The University of Tokushima Graduate School, Kuramoto, Tokushima 770-8504, Japan

**Keywords:** tumor necrosis factor-α, interleukin-34, nuclear factor-κB, osteoblast

## Abstract

Osteoblasts produce various types of cytokines under pathological conditions and control osteoclast differentiation. Tumor necrosis factor-α (TNF-α) has been demonstrated to exert complex effects in osteoblasts under local inflammatory conditions, including in periodontal and periapical diseases. Interleukin-34 (IL-34) has been recently identified as a novel regulatory factor for the differentiation and function of osteoclasts. The present study provides the first evidence, to the best of our knowledge, that the expression of IL-34 is induced by TNF-α through nuclear factor-κB (NF-κB) activation in MC3T3-E1 osteoblastic cells. TNF-α induced IL-34 expression in a dose- and time-dependent manner. Immunocytochemistry with an NF-κB antibody demonstrated that NF-κB was mainly localized in the cytoplasm of the untreated MC3T3-E1 cells. Rapid translocation of NF-κB from the cytoplasm to the nucleus was observed in the cells treated with TNF-α for 15 min. Translocation and transcriptional activity of NF-κB were also determined by western blotting and a luciferase reporter assay, respectively. Pretreatment with 100 μM CAPE, an inhibitor of NF-κB, significantly inhibited TNF-α-induced IL-34 expression. These results indicate that TNF-α induces IL-34 expression via NF-κB in osteoblasts.

## Introduction

Skeletal systems are maintained by continuous bone remodeling, which is a process regulated by osteoblasts and osteoclasts. Osteoblasts have a critical role in bone formation and re-modification by producing stimulatory and inhibitory factors that tightly regulate osteoclast formation and activity. The functioning of osteoblasts is regulated by numerous factors including hormones, growth factors and cytokines, such as tumor necrosis factor-α (TNF-α) (1). TNF-α is synthesized in the bone microenvironment and has been demonstrated to exert pleiotropic effects on osteoblasts. High levels of TNF-α have been identified in the tissue and gingival crevicular fluid of patients with advanced periodontitis and chronic periapical periodontitis (2–4). Increased TNF-α levels have also been identified in primarily infected root canals and the infected periapical tissue of patients with pulpitis and chronic periapical periodontitis (5,6). Therefore, the expression of TNF-α is considered to be correlated with the progression of bone resorption in periodontal and periapical diseases.

TNF-α has been reported to stimulate the production of macrophage colony stimulating factor (M-CSF) in osteoblasts (7,8), which binds to M-CSF receptor (CSF-1R) on the surface of pre-osteoclasts. This binding stimulates osteoclast differentiation and alveolar bone resorption (9,10). Previously, interleukin-34 (IL-34) was identified as a novel cytokine with similar characteristics to M-CSF (11). Although IL-34 shares no sequence homology with M-CSF, IL-34 binds to the CSF-1R and promotes the differentiation, proliferation and survival of osteoclasts. It was also reported that IL-34 as well as M-CSF, in combination with receptor activated nuclear factor-κB (NF-κB) ligand (RANKL), promoted osteoclast differentiation and bone resorption in mouse and human cell culture systems (12). However, whether TNF-α induces IL-34 expression in mouse osteoblasts has not yet been fully investigated.

Nuclear factor-κB (NF-κB) is a transcription factor, which is activated by numerous types of extracellular stimuli, including bacterial products, oxidative stress and physical stress. NF-κB is a multifunctional transcription factor that regulates various gene expression involved in numerous cellular activities (13,14). In unstimulated cells, NF-κB is sequestered in the cytoplasm bound to nuclear factor of κ light polypeptide gene enhancer in B-cell inhibitors (IκBs). When NF-κB activation is stimulated, IκB is phosphorylated, ubiquitinated and then degraded by the protease, thereby allowing translocation of the liberated NF-κB from the cytoplasm to the nucleus (15). NF-κB is activated in numerous inflammatory conditions. Activated nuclear NF-κB, in turn, regulates the expression of cytokines and so mediates autocrine, self-amplifying cycles of cytokine release (13,14). Several studies have demonstrated that TNF-α induces NF-κB activation in human or rat osteoblastic cells as well as osteoclasts, which mediates the upregulation of interleukin-6 (IL-6) and intercellular adhesion molecule-1 (ICAM-1) (16,17). However, whether NF-κB activation induced by TNF-α is involved in IL-34 expression in mouse osteoblasts remains elusive.

In the present study, the expression of IL-34 in TNF-α-treated mouse MC3T3-E1 osteoblastic cells was examined. The involvement of NF-κB in TNF-α-induced IL-34 expression in osteoblasts was also investigated.

## Materials and methods

### Materials

α-modified Eagle’s minimal essential medium (α-MEM), OPTI-MEM and Lipofectamine™ reagent were purchased from Invitrogen Life Technologies (Carlsbad, CA, USA); TNF-α was purchased from Sigma-Aldrich (St. Louis, MO, USA); antibodies against NF-κB and Eps were obtained from Santa Cruz Biotechnology, Inc. (Santa Cruz, CA, USA); anti-lamin B1 monoclonal antibody was from Zymed (South San Francisco, CA, USA) and caffeic acid phenethyl ester (CAPE) were purchased from Biomol International (Philadelphia, PA, USA).

### Cell culture

The MC3T3-E1 mouse osteoblastic cells (RIKEN Cell Bank, Tsukuba, Japan) were cultured in plastic dishes containing α-MEM supplemented with 10% FBS at 37°C in a humidified atmosphere of 5% CO_2_ and 95% air. The cells were subcultured every three days by treating the cells with 0.25% trypsin together with 1 mM EDTA in Ca^2+^-, Mg^2+^-free phosphate-buffered saline (PBS). For immunofluorescence, MC3T3-E1 cells were grown on sterile 18-mm round glass coverslips and cultured for the desired periods.

### RNA preparation and quantitative polymerase chain reaction (qPCR) assay

Following the appropriate treatment, total cellular RNA was isolated from MC3T3-E1 cells using RNAiso Plus (Takara Bio, Inc., Shiga, Japan), followed by phenol extraction and ethanol precipitation. The purified RNA was further incubated with DNase I (Sigma-Aldrich) to digest the contaminating DNA. cDNA was synthesized using ReverTra Ace^®^ qPCR RT Master mix (Toyobo, Tokyo, Japan). qPCR was conducted using SYBR^®^ Select Master mix (Applied Biosystems, New York, NY, USA). Amplified reactions were quantified on an ABI 7500 real-time PCR system (Applied Biosystems). Relative gene quantities were obtained using the comparative Ct method following normalization to the appropriate control genes (β-actin). qPCR was performed on the cDNA with the following primers: Forward: 5′-CTTTGGGAAACGAGAATTTGGAGA-3′ and reverse: 5′-GCAATCCTG TAGTTGATGGGGAAG-3′ for mouse IL-34; and forward: 5′-CAATAGTGATGACCTGGCCGT-3′ and reverse: 5′-AGAGGGAAATCGTGCGTGAC-3′ for mouse β-actin.

### Immunocytochemistry

Cells were grown on sterile 18-mm round glass cover slips placed in 60-mm plastic dishes and treated with 0, 1 and 10 ng/ml TNF-α for 0, 15, 30 and 60 min. Additionally, cells were pretreated with 100 μM CAPE for 1 h and treated with 10 ng/ml TNF-α for 15 min. The coverslips were washed three times with PBS and fixed with 3.7% formaldehyde for 10 min at ambient temperature, followed by methanol-permeabilization for an additional 20 min at −20°C. Non-specific binding sites were blocked with 4% bovine serum albumin (BSA) in PBS for 20 min at ambient temperature. The coverslips were incubated with anti-p65 NF-κB antibody diluted 1:500 for 45 min at ambient temperature. The cells were then incubated for 30 min with Alexa 488-conjugated goat anti-rabbit IgG (Invitrogen Life Technologies) diluted 1:500 in 4% BSA in PBS, followed by incubation for 15 min with 10 μg Hoechst 33342 diluted 1:500 for nucleus staining at ambient temperature. The coverslips were washed with PBS and mounted with fluorescent mounting medium (DakoCytomation, Carpinteria, CA, USA). The samples were examined under an Olympus BX50 microscope (Olympus, Tokyo, Japan) equipped with epifluorescence illumination. Photomicrographs were recorded on a computer (DP70-WPCXP; Olympus).

### Fractionation of the nucleus and cytosol, and western blot analysis

Following treatment with 10 ng/ml TNF-α for 0 and 15 min, cells cultured in 90-mm plastic dishes were washed twice with PBS, collected and resuspended in hypotonic buffer (20 mM HEPES, pH 7.2; 10 mM KCl, 1 mM MgCl_2_, 1 mM DTT and 0.5 mM EDTA). Cells were allowed to swell for 10 min in ice prior to lysis by addition of 0.1% NP-40 and 100 mM potassium acetate. Following 5 min incubation on ice, the cytosolic fraction was recovered in the supernatant after centrifugation (10,000 × g for 5 min at 4°C). The pelleted nuclei were resuspended in lysate buffer containing 1 mM DTT, 1 mM PMSF, 1 mg/ml leupeptin, 2 mg/ml aprotinin and 5 mM EGTA in PBS, and following centrifugation (20,000 × g for 10 min at 4°C). The nuclear fraction was recovered in the supernatant. The protein concentration of each sample was evaluated using the protein assay reagent (Bio-Rad, Hercules, CA, USA). A total of 12 μg of each sample and prestained molecular weight markers were separated by SDS-PAGE and transferred to polyvinylidene fluoride membranes (Immobilon-P; Millipore, Bedford, MA, USA). The membranes were blocked in 5% skimmed milk in PBS-Tween-20 for 2 h. The membranes were incubated in PBS-Tween-20 containing anti-p65 NF-κB antibody (diluted at 1:1,000) overnight at 4°C followed by incubation for 2 h at ambient temperature with an anti-rabbit horseradish peroxidase-linked secondary antibody (diluted at 1:5,000; Cell Signaling Technology, Inc., Danvers, MA, USA). The reaction was visualized with an enhanced chemiluminscence detection kit (GE Healthcare, Chalfont, UK) according to the manufacturer’s instructions.

### Dual-luciferase reporter assay

The luciferase plasmid pNF-κB-Luc was obtained from Stratagene (La Jolla, CA, USA). MC3T3-E1 cells were seeded into 35-mm plates at a density of 2.0×10^5^ cells/well. Following 24 h, the cells were co-transfected with 1 μg of pNF-κB-Luc and 0.05 μg pRL-TK *Renilla* luciferase vector (Promega Corporation, Madison, WI, USA) with the aid of the Lipofectamine reagent. Following 24 h the cells were treated with or without TNF-α for the indicated duration. The cells were harvested and treated with passive lysis buffer according to the dual-luciferase assay manufacturer’s instructions (Promega Corporation). The signals of firefly luciferase activity were normalized with respect to pRL-TK *Renilla* luciferase signals for individual analysis to eliminate the variations of transfection efficiencies. Data were analyzed by analysis of variance (ANOVA) and Bonferroni/Dunn’s test was utilized to estimate the significance between the means.

### Statistical analysis

Each series of experiments were repeated at least three times and the data are expressed as mean values ± standard error of mean. Statistical analysis was performed by ANOVA. P<0.05 was considered to indicate a statistically significant difference.

## Results

### TNF-α increases IL-34 mRNA expression in a dose- and time-dependent manner in MC3T3-E1 cells

**Figure 1:**
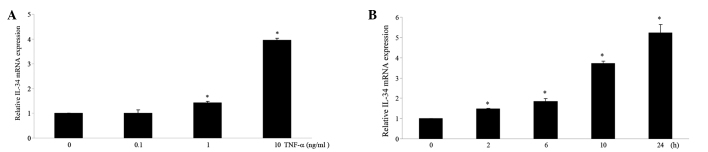
TNF-α increased IL-34 mRNA expression in a dose- and time-dependent manner in MC3T3-E1 cells. (A) MC3T3-E1 cells were treated with various doses of TNF-α for 10 h. The expression of IL-34 mRNA was determined by qPCR. (B) MC3T3-E1 cells were treated with 10 ng/ml TNF-α for the indicated time periods. The expression of IL-34 mRNA was determined by qPCR. ^*^P<0.05, compared with the control group. TNF-α, tumor necrosis factor-α; IL-34, interleukin-34; qPCR, quantitative polymerase chain reaction.

To examine the effect of TNF-α on IL-34 mRNA expression in mouse osteoblastic cells, MC3T3-E1 cells were treated with different doses of TNF-α. RNA was collected from the treated cells and subjected to qPCR using specific primer pairs as indicated in the Materials and methods. Treatment with TNF-α increased IL-34 mRNA expression in a dose-dependent manner (Fig. 1A). The expression of IL-34 mRNA was also increased in a time-dependent manner by TNF-α treatment (Fig. 1B).

### TNF-α induces translocation and activation of NF-κB in MC3T3-E1 cells

**Figure 2:**
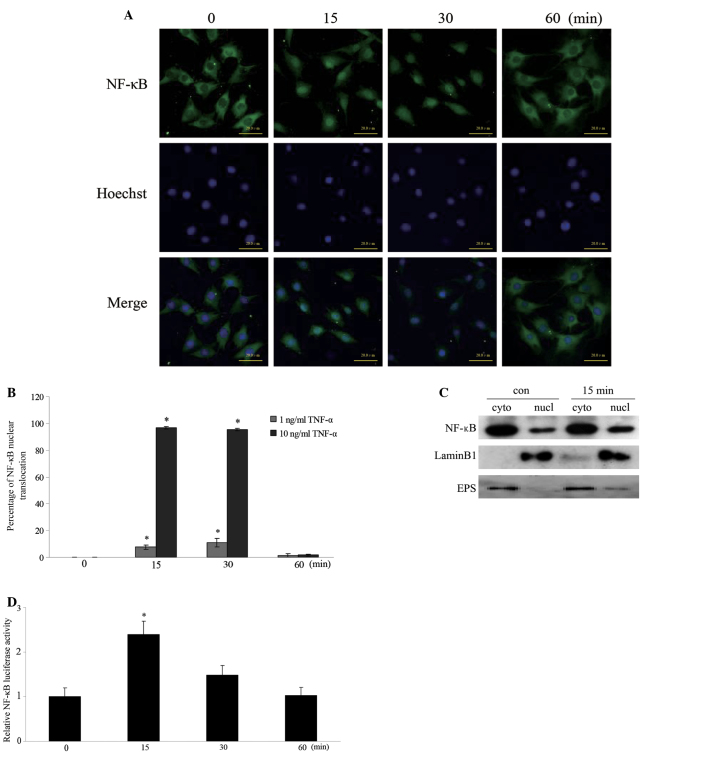
TNF-α induces the translocation and activation of NF-κB in MC3T3-E1 cells. (A) MC3T3-E1 cells were treated with 10 ng/ml TNF-α for the indicated times. The cells were subjected to indirect immunofluorescence with anti-NF-κB antibody (top panel). The same samples were incubated with Hoechst 33342 (middle panel) for nuclear staining and the images were merged (bottom panel). (B) MC3T3-E1 cells were treated with various doses of TNF-α for the indicated times. Following immunofluorescence with anti-NF-κB antibody, the percentages of nuclear translocation of NF-κB were counted. ^*^P<0.05, compared with the control group. (C) The cells were treated with 10 ng/ml TNF-α for 15 min. The cytosolic and nuclear fractions were subjected to SDS-PAGE with subsequent western blot analysis with antibodies against NF-κB, Eps and Lamin B1. (D) MC3T3-E1 cells were transfected with NF-κB-Luc or pGL3 (empty vector). Following 24 h, the cells were treated with 10 ng/ml TNF-α for the indicated times. Then, the cells were lysed with passive lysis buffer and the luciferase activity was measured. The results were normalized with respect to pRL-TK activities. Each column represents the mean ± SD. ^*^P<0.05, compared with the control group. TNF-α, tumor necrosis factor-α; IL-34, interleukin-34; NF-κB, nuclear factor-κB.

To examine whether TNF-α treatment altered the subcellular localization of NF-κB, MC3T3-E1 cells were incubated with 10 ng/ml TNF-α for 0, 15, 30 and 60 min. Fig. 2A demonstrates that NF-κB was mainly localized in the cytoplasm in the untreated cells. Rapid translocation of NF-κB into the nucleus was observed in the cells treated with TNF-α for 15 and 30 min. Fig. 2B reveals the percentages of nuclear translocation of NF-κB in the cells treated with 1 and 10 ng/ml TNF-α. The percentages of nuclear translocation of NF-κB treated with 1 ng/ml TNF-α for 15 and 30 min were 7.6±1.59 and 11.3±3.16%, respectively. However, the percentages of nuclear translocation of NF-κB treated with 10 ng/ml TNF-α for 15 and 30 min were 96.6±0.88 and 95.4±0.90%, respectively. To further determine whether TNF-α induced NF-κB translocation, cell fractionation was performed using the cells treated with 10 ng/ml TNF-α for 15 min. Fig. 2C demonstrates that the intensity of the band corresponding to NF-κB in the nuclear fraction was increased following TNF-α treatment for 15 min compared with that of the unstimulated cells. The purity of nuclear and cytosolic fractions was confirmed using an antibody against Lamin B1 (middle) and anti-Eps15 antibody (bottom), respectively. To further examine whether TNF-α regulates NF-κB transcriptional activity, the luciferase reporter assay was performed. TNF-α treatment for 15 min increased the luciferase activity >2-fold compared with that of the control cells (Fig. 2D). These results indicate that TNF-α stimulates NF-κB nuclear translocation and transcriptional activity.

### TNF-α increases IL-34 expression via the NF-κB-dependent pathway in MC3T3-E1 cells

**Figure 3:**
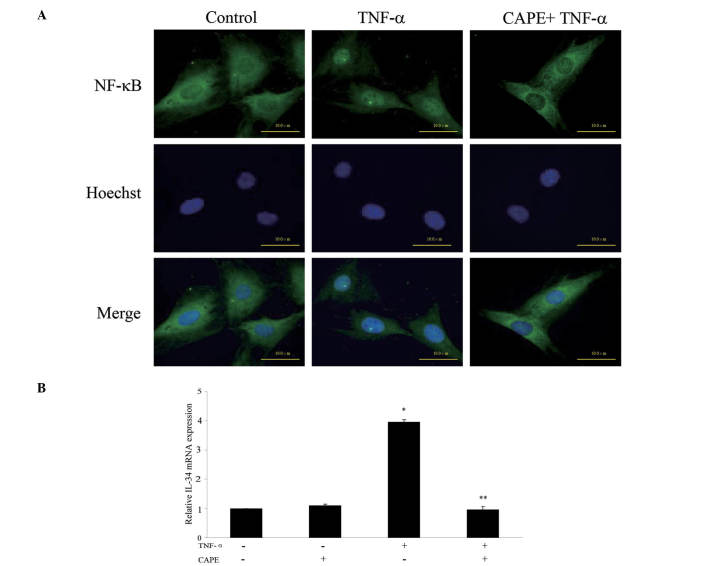
TNF-α regulated expression of IL-34 mRNA via the NF-κB-dependent pathway in MC3T3-E1 cells. (A) MC3T3-E1 cells were pretreated with 100 μM CAPE for 1 h and treated with 10 ng/ml TNF-α. Then, the cells were subjected to indirect immunofluorescence with anti-p65NF-κB antibody. (B) MC3T3-E1 cells were pretreated with 100 μM CAPE for 1 h and treated with 10 ng/ml TNF-α. The expression of IL-34 mRNA was determined by qPCR. ^*^P<0.05, compared with the control group. ^**^P<0.05, compared with the TNF-α treated group. TNF-α, tumor necrosis factor-α; IL-34, interleukin-34; NF-κB, nuclear factor-κB; qPCR, quantitative polymerase chain reaction.

To examine whether NF-κB is involved in the IL-34 expression induced by TNF-α, the cells were treated with TNF-α following pretreatment with CAPE, an inhibitor of NF-κB. The nuclear translocation of NF-κB induced by TNF-α was inhibited by CAPE (Fig. 3A). Pretreatment with 100 μM CAPE for 1 h significantly inhibited TNF-α-induced IL-34 expression (Fig. 3B). However, treatment with CAPE alone did not change the TNF-α-induced IL-34 expression (Fig. 3B).

## Discussion

In the present study, the effect of TNF-α on IL-34 expression and NF-κB activation in osteoblasts was examined. It was demonstrated that TNF-α induced IL-34 expression in MC3T3-E1 cells and NF-κB was involved in the TNF-α-induced IL-34 expression in these cells.

Experimental and clinical studies have demonstrated that TNF-α is an important factor for bone resorption resulting from periodontal and periapical diseases (2–6,18). Elevated TNF-α levels in infected root canals, gingival crevicular fluid and saliva of patients with aggressive periodontitis has been reported to stimulate osteoclast generation through the induction of M-CSF (8,19). Although IL-34 was demonstrated to have a similar function to M-CSF, the correlation between TNF-α and the expression of IL-34 in osteoblasts has not been clarified. In the present study, it was demonstrated that TNF-α treatment induced IL-34 expression in MC3T3-E1 cells in a dose- and time-dependent manner. IL-34 is a novel cytokine, which binds to the M-CSF receptor and possesses similar characteristics to M-CSF in promoting monocyte viability and osteoclast generation (11,12). These observations suggest that IL-34 produced from osteoblasts stimulates osteoclast generation, which leads to bone resorption in the inflammatory regions.

Following this, the present study aimed to clarify the mechanism of IL-34 expression induced by TNF-α treatment in osteoblasts. It was demonstrated that TNF-α treatment rapidly induced NF-κB translocation from the cytoplasm into the nucleus in MC3T3-E1 cells, as determined by immunostaining and cell fractionation assays. Consistent with these results, the luciferase assay revealed that NF-κB transcriptional activity was significantly increased in MC3T3-E1 cells treated with TNF-α. It was reported that TNF-α induced M-CSF expression in primary osteoblasts and osteoblastic cells through the activation of NF-κB (20,21). Therefore, it was hypothesized that NF-κB is involved in the IL-34 expression in MC3T3-E1 cells induced by TNF-α. To verify this hypothesis, MC3T3-E1 cells were pretreated with NF-κB specific inhibitor CAPE, followed by TNF-α treatment for 15 min. Pretreatment of 100 μM CAPE markedly inhibited TNF-α-induced IL-34 expression in MC3T3-E1 cells, indicating that NF-κB is involved in the expression of IL-34 in these cells. Previously, it was reported that TNF-α-induced IL-34 expression in synovial fibroblasts was mediated through the activation of NF-κB (22,23). NF-κB mediates the expression of numerous inflammatory genes, including IL-6 and ICAM-1, in rat and human osteoblast-like cells, such as UMR106 and MG63 cells (16,17,24,25). By contrast, it was reported that lipopolysaccharide and interferon-γ only moderately increased IL-34 mRNA levels, although these factors were well-known stimuli for eliciting a variety of inflammatory responses (26). The present results, coupled with the previous evidence, suggests that NF-κB is an important mediator for regulating the expression of inflammatory cytokines induced by TNF-α in osteoblastic cells.

In conclusion, the data demonstrated in the present study provides evidence that TNF-α induces IL-34 expression via NF-κB in MC3T3-E1 cells. Further investigation is required to define the pathological implications of IL-34 produced from osteoblasts in local inflammatory regions, including in periodontal and periapical diseases. Studies designed to examine the potential of the NF-κB pathway as a therapeutic target for these inflammatory conditions *in vivo* are also warranted.
